# Outlook on the Application of Metal-Liganded Bioactives for Stimuli-Responsive Release

**DOI:** 10.3390/molecules22122065

**Published:** 2017-11-26

**Authors:** Gretta C. M’bitsi-Ibouily, Thashree Marimuthu, Pradeep Kumar, Lisa C. du Toit, Yahya E. Choonara, Pierre P. D. Kondiah, Viness Pillay

**Affiliations:** Wits Advanced Drug Delivery Platform Research Unit, Department of Pharmacy and Pharmacology, School of Therapeutic Sciences, Faculty of Health Sciences, University of the Witwatersrand, Johannesburg, 7 York Road, Parktown 2193, South Africa; 362179@students.wits.ac.za (G.C.M.-I.); thashree.marimuthu@wits.ac.za (T.M.); pradeep.kumar@wits.ac.za (P.K.); lisa.dutoit@wits.ac.za (L.C.d.T.); yahya.choonara@wits.ac.za (Y.E.C.); pierre.kondiah@wits.ac.za (P.P.D.K.)

**Keywords:** metal-liganded bioactive, drug carrier, stimuli-responsive release, bioinorganic medicine

## Abstract

Direct metal-liganded bioactive coordination complexes are known to be sensitive to stimuli such as pH, light, ion activation, or redox cues. This results in the controlled release of the bioactive(s). Compared to other drug delivery strategies based on metal complexation, this type of coordination negates a multi-step drug loading methodology and offers customized physiochemical properties through judicious choice of modulating ancillary ligands. Bioactive release depends on simple dissociative kinetics. Nonetheless, there are challenges encountered when translating the pure coordination chemistry into the biological and physiological landscape. The stability of the metal–bioactive complex in the biological milieu may be compromised, disrupting the stimuli-responsive release mechanism, with premature release of the bioactive. Research has therefore progressed to the incorporation of metal-liganded bioactives with established drug delivery strategies to overcome these limitations. This review will highlight and critically assess current research interventions in order to predict the direction that pharmaceutical scientists could pursue to arrive at tailored and effective metal-liganded bioactive carriers for stimuli-responsive drug release.

## 1. Introduction

Bio-inspired metal coordination has demonstrated a growth in applications in the field of drug delivery [1,2,3]. Recent reports have highlighted the release of the drug cargoes through the dissociation of bespoke metal–ligand bonds [4,5,6] or metal–biomolecule bonds [7]. An alternative approach to these methodologies is the direct coordination of the bioactive to the metal center, which results in metal-liganded bioactive complexes. These complexes are advantageous as they are capable of delivering the bioactive through selective release mechanism(s) [8]. Metal–ligand bioactives in the context of this review refer to the coordination of a market-available pharmaceutical agent to a metal center to produce a novel and more effective drug delivery system. Notably, the introduction of the metal center can control the mechanism by which the drug is delivered without changing the drug action or therapeutic activity. Furthermore, this is a relatively more facile route to enhancing both the pharmacokinetic and pharmacodynamic properties of the parent bioactive.

Approximately 60–70% of drug molecules suffer from poor aqueous solubility and/or low permeability following oral delivery [9]. The coordination of drugs to metal centers is a promising approach that has the potential to improve both the aqueous and lipid solubility of poorly absorbed bioactives [10]. The increase in lipid solubility has been primarily attributed to chelation effects, which results in a delocalization of electron density among the donors of the bioactive and/or ancillary ligand. This has been reported to increase the lipophilic character of the metal-liganded bioactive, and promotes permeation through the lipid layers of the target cell membranes [11,12]. The corresponding improvement in aqueous solubility can be attributed to the inherent hydrophilic nature of metals; for example, bis(esomeprazole) magnesium complexes are relatively more soluble than the parent drug, esomeprazole [13]. In addition, oral bioavailability can be improved if a drug is formulated with a delivery system that has the ability to release substrates that inhibit the back-efflux process [14]. An example of drug efflux in the gastrointestinal tract is the ability of P-glycoprotein to pump drugs back into the lumen, decreasing absorption and lowering bioavailability. The metal complex itself (or upon functionalization with ancillary ligands) could potentially inhibit this process, thereby increasing absorption. Drug solubilization by complexation through non-covalent interactions can also be achieved through the preparation of protein-liganded bioactives [15]. A recent report has shown the use of natural proteins such as serum albumin for complexation to rifapentine [16], which resulted in improved oral bioavailability of rifapentine coupled with relative improved stability of the resulting protein-liganded rifapentine solutions. However, other protein-liganded drug complexes are generally more susceptible to quick degradation [16] and in these instances metal complexation provides more stable complexes due to formation of bonds through e^−^ sharing compared to covalent interactions. Furthermore, proteins have specific available donors based within their network and these donor sites are arranged randomly within the protein network; therefore, specificity to the bioactive is low and early bioactive release is possible in the presence of other competing drugs. On the contrary, metal complexation offers more control as a metal center can be chosen based on the donors available on the bioactive and ligands, and the coordination sphere around the metal sphere results in a spatial arrangement of bioactive and other ligands emanating in a defined geometry. The effects of metal complexation are therefore uniformly distributed over the coordination sphere and this offers a further opportunity to tailor the properties of the metal-liganded complex.

Alternatively, and perhaps a more ingenious opportunity, lies in the fact that metal-liganded bioactive coordinative bonds are responsive to environmental stimuli, e.g., pH, redox potential, or localized application of light. Therefore, a research niche exists for the development of controlled-through-stimuli-triggered bioactive release systems enabled by metal complexation. This smart drug delivery biomaterial approach [17] can result in lower systemic toxicity. A more specific example is the complex [Cu(fen)_2_(im)_2_], where Cu = copper(II) ion; fen = fenoprofenate anion from Fenoprofen (bioactive); and im = imidazole (ancillary ligand), with the overall complex reported to have enhanced analgesic activity and reduced ulcerogenic action compared to the free drug, fenoprofen [18]. To date, stimuli-responsive metal-liganded bioactive systems have found application in the release of small molecules such as CO_2_, and NO [19,20] but the delivery of relatively larger bioactives is scant [21,22,23].

Moreover, there has been a recent review on the current application of metal-liganded bioactives for use in drug delivery [8]; however, this review did not focus on additional formulation aspects that could further control bioactive release and also protect the metal complex during oral delivery. This paper will therefore highlight recent examples of metal-liganded bioactive complexes where (a) the bioactive can form one or more coordinative bonds with the metal center though available donor sites; (b) the bioactive may belong to, but is not limited to, an anti-cancer, anti-inflammatory, anti-microbial, or neuroactive drug class; (c) the complex demonstrates stimuli-responsive bioactive release; and (d) additional formulation strategies have been employed.

The route of delivery of the intended metal-liganded bioactive has been established as an important factor that could influence the selective release of the bioactive, and in most instances further formulation is required. For example, a copper-liganded naproxen (Npx) complex, [Cu_2_(Npx)_4_(dmso)_2_], was previously prepared and loaded into chitosan beads for the oral delivery of naproxen [23]. Thereafter, a portion of the complex-loaded beads were coated with a methacrylic acid co-polymer for reducing naproxen release at gastric pH (from 55 to 20%).

Further, the preparation of two zinc metal–drug complexes based on theophylline (TPL) [22], viz. [Zn(TPL)_2_(H_2_O)_2_] and [Zn_2_(TPL)(FA)(OH)(H_2_O)], where FA = fumaric acid (ancillary ligand), were reported by Wang et al. [21]. These complexes were encapsulated in a polymer matrix comprising of hydroxypropyl methylcellulose (HPMC) and microcrystalline cellulose (MCC). Relative to the control experiment, the metal-liganded formulations demonstrated slower drug release and the rate of release was controlled by varying the amount of MCC added, with the release mechanism varying from inconsistent delivery (free drug) to controlled and steady Fickian diffusion [22]. To this end, this review will also examine research employing sophisticated applications that incorporate the metal coordination strategy with additional cutting edge formulation protocols in order to maintain the controlled stimuli-responsive metal-liganded bioactive delivery mode and prevent premature release of the bioactive.

## 2. pH-Triggered Bioactive Release

### 2.1. Bioinspired Materials

**Figure 1:**
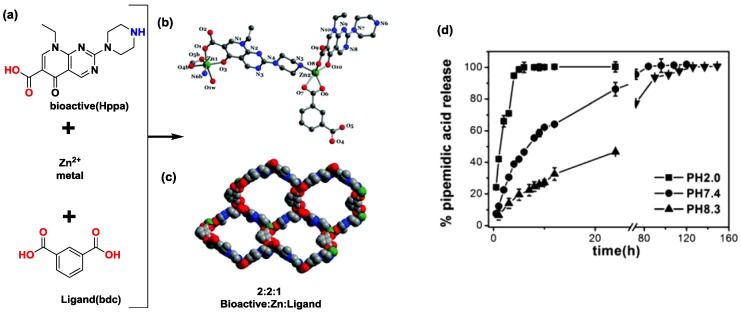
(**a**,**b**) Graphical representation based on X-ray single-crystal diffraction data of complexation between Zn ions (green), with O donor in Hppa, O donors in bdc (red) with another Zn ion with N donor in Hppa (blue); (**c**) The structure of the MOF resembles a 2D interpenetrated network with 6-membered rings; (**d**) Reported release profile of Hppa from MOF. Reproduced with permission from the Royal Society of Chemistry [26].

Metal biomolecule frameworks (bioMOFs) are fabricated using endogenous molecules or bioactives as structural entities [24]. This synthetic design endows the framework with minimal toxicity concerns and/or minimizes the amount of bridging or ancillary ligands required. Moreover, this methodology negates entrapment of the bioactive via impregnation methods that lead to the two-fold advantage of (a) a high drug loading capability [25] and (b) controlled release systems. The latter design is due to the fact that the interpenetrated metal-liganded bioactive bonds allow for slower release of the bioactive in physiological media [2]. An example of this behavior was observed for a novel MOF, with formula [Zn_2_(ppa)_2_(1,3-bdc)(H_2_O)]·2H_2_O, where ppa = pipemidic acid (bioactive), and bdc = 1,3-benzenedicarboxylate (low toxic ancillary ligand) [26] (Figure 1). The ambidentate nature of pipemidic acid and bidentate properties of bdc are the structural building blocks for the generation of the MOF (Figure 1a–c) The corresponding in vitro release kinetics of Hppa from the MOF with particle size distribution of 25–40 μm was recorded in simulated gastric fluid (SGF, pH = 2), body fluid (SBF, pH = 7.4) and intestinum crassum fluid (SIC, pH = 8.3), respectively (Figure 1d). The results depict a pH-responsive delivery of Hppa, with controlled release (38.1%) over 48 h observed at the highest pH values tested. It was reported that the significant release rate in SGF is due to the free protons competing for donor sites on pipemidic acid, which resulted in MOF degradation and evident drug release. Nonetheless, this limitation could be rectified through possible additional formulation steps. As a nascent field, future research endeavors should consider the investigation of in vivo stimuli-triggered degradation efficiency, and cell culture studies should be undertaken to verify the biocompatibility of the system. Possible considerations should also include the sole use of biomolecules as ancillary or pillaring ligands for production of the MOF to ensure overall improved biocompatibility.

**Figure 2:**
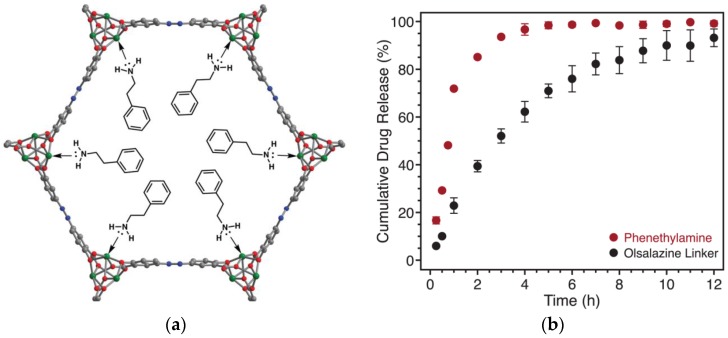
(**a**) Coordination between phenethylamine and available sites of Mg^2+^. The illustrated model is proposed from X-ray powder diffraction data, where green, red, blue, and gray spheres represent Mg, O, N, and C atoms, respectively. The hydrogen atoms have been removed for clarity; (**b**) The observed release of both phenethylamine and olsalazine from the MOF at in phosphate buffer medium of pH 7.4, at 37 °C. Reproduced from with permission from the American Chemical Society (2016) [27].

A bioMOF with remarkable biocompatibility was exemplified by the application of magnesium-liganded olsalazine bioMOF (Figure 2a) for the co-delivery of olsalazine and entrapped phenethylamine [27]. Notably, the BioMOF achieved an unsurpassed loading of 86 wt % drug in the framework. The bioMOF was then assessed for in vitro release capabilities and it was reported that under physiological conditions of pH 7.4 that the respective release of phenethylamine and olsalazine was 95 and 50% after 3 h (Figure 2b). The mechanism of release of olsalazine was much slower due to the nature of the interpenetrated Mg-liganded olsalazine bonds further highlighting the benefit of employing this bioMOF design. As the system has already shown merited biocompatibility, forthcoming research endeavors should consider tailoring the release mechanism, followed by in vivo verification of the drug delivery performance.

Another example of a bioinspired drug delivery system involved the preparation of Fe-liganded doxorubicin (DOX) formulated into transferrin-inspired nanoparticles (NPs) [28]. The pH-triggered release of DOX was suitable for intracellular release at the acidic tumor site. This was evident in the release of 73.6% and 59.3% of DOX observed at pH 4.5 and 5.5, respectively, which simulated the conditions of the tumor microenvironment. The in vitro release at pH 6.5 (extracellular tumour, milieu) and 7.4 was 33.5% and 25.6%, respectively [28]. The pH-responsive Fe-liganded DOX bond cleave was also verified through in vivo studies using a mouse model through intravenous administration of the prepared NPs.

Furthermore, the introduction of the ferric ion was beneficial to the rational design of the biomimetic drug delivery system (BDDS) in the following conditions: (a) higher loading of DOX was obtained for Fe formulations as compared to the control NPs; (b) the ferric ion also served as a crosslinking agent to increase the stability of Tf-inspired NPs; and (c) the ferric ion coordination sphere was large enough to also promote self-assembly for the formation of the Tf-inspired NPs through coordinative bonds [28]. The use of metal coordination in this study demonstrates the versatility of metal ions and such smart delivery systems could also be extended to targeted delivery applications by employing the metal as a linker for antibody drug-based conjugates.

### 2.2. Self-Assembled Metal-Liganded Bioactives

The complexation of Minocycline Hydrochloride (MH) with Ca(II) and or Mg(II) ions in the presence of dextran sulphate (DS) was undertaken. This rational design involved the metal cations in the metal-liganded MH complex, enabling ionic interactions with the negative donors on the DS polysaccharide backbone to fabricate a DS-metal ion-MH complex [29]. The metal-liganded MH chelates were stable at pH 7.5; however, at pH 6.0 the drug release was more significant. Sustained release under pathology-induced tissue acidosis was observed over 71 days as the metal-liganded MH complexes have strong coordinative bonds that can result in controlled bioactive release. This system could easily be applied to other bioactives; however, these bioactives must have a high affinity to bind to the metal centers and also must have more than one donor available for coordination in order to promote chelate formation, which will facilitate controlled drug release.

**Figure 3:**
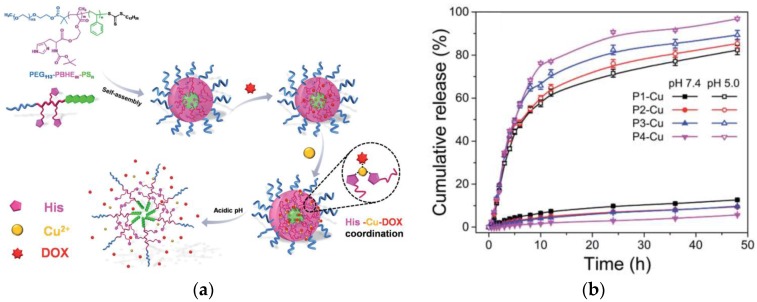
(**a**) Scheme depicting drug loading into PEG113-PBHEm-PSn micelles promoted by the metal coordination of copper to both doxorubicin and histidine followed by (**b**) pH-responsive release of the bioactive. Reproduced with permission from the Royal Chemical Society [31].

Bai and co-workers established a coordination interaction occurring at a neutral pH between metal ions (e.g., Cu^2+^) and the common anticancer drug, DOX, with dissociation in acidic environments, thus potentially enabling precise drug release in cancer cells [30]. Another group of pH-responsive assemblies thus includes polymeric micelles formulated with poly(ethylene glycol)-*b*-poly(2-hydroxyethylmethacrylate-Boc-histidine)-*b*-poly(styrene) (PEG-PBHE-PS) with metal-liganded bioactive complexation (Figure 3a) [31]. In this system, copper was coordinated to both the biocompatible ancillary ligand, histidine, and to the bioactive DOX, and loadings up to 47.4% of DOX were obtained as compared to 12% for control micelles (with no metal coordination present). The metal complexation strategy therefore promoted the drug loading ability of this fabrication methodology. The release of DOX at a lower pH of 5 was more pronounced than at a higher pH of 7.4 (Figure 3b), and it was concluded that coordinative bond cleavage between metal and bioactive was the rate-determining step for drug release as opposed to simple diffusion kinetics [30,32]. The improved drug loading and smart intracellular delivery warrant further analysis of this system via in vivo experiments to verify the therapeutic efficacy.

## 3. Light-Triggered Bioactive Release

### Ruthenium Cage Complexes

**Scheme 1:**
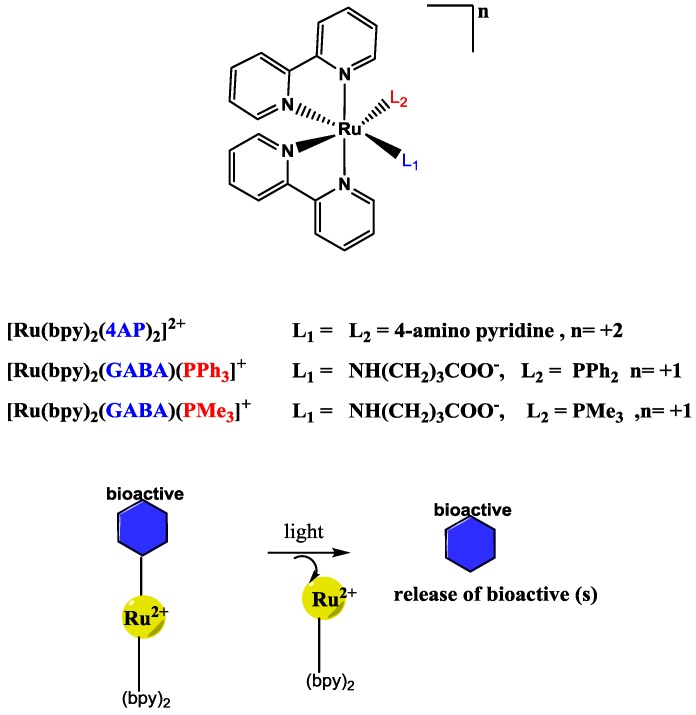
Ru(II) polypyridyl complexes that release neuroactives upon light irradiation.

Ruthenium (Ru) metal centers have been reported for the preparation of octahedral Ru(II) polypyridyl complexes, which release neuro-actives upon light irradiation [32]. The coordination chemistry of octahedral Ru(II) polypyridyl complexes offers the possibility of caging an extended range of molecules, including amino acids, nucleotides, neurotransmitters, and fluorescent probes [32]. In addition, these Ru-based caged compounds are responsive to visible light, unlike other phototriggers, and can be photolyzed employing green light. These characteristics enable the use of simple and inexpensive equipment [32]. Ru(II) polypyridyl complexes are also active in the two-photon regime, a property that widens their scope to systems that use infrared light to achieve high precision and penetrability [32]. These complexes were reported to release 4-aminopyridine (4AP) [33] and γ-aminobutyric acid (GABA) [34]. The design of these complexes incorporates the inherent photo-physical properties of the Ru(II) polypyridyl cation, and the photo-responsive cleavage of the weaker [metal-bioactive bond] to release the bioactive. The reported complex containing Ru-linked 4-AP was obtained at a good yield using a simple one-step synthetic approach and demonstrated successive controlled release of the two molecules of 4AP molecules incorporated, evidenced by Nuclear Magnetic Resonance (NMR) studies [33]. The corresponding biological activity was observed by the activation of neuronal firing in a leech ganglion [33]. Similarly, the complex RuBi-GABA (Scheme 1) was reported to release GABA;,coupled with high tissue penetration, less phototoxicity, and faster photo-release kinetics compared to other UV light- sensitive caged compounds [34]. In vitro tests demonstrated no effect on endogenous GABAergic or glutamatergic transmission at concentrations effective for measurable release. In a related in vivo study (mouse cortex), photo-release of GABA from a new derivative indicated that high formulation doses were tolerable [35].

Ru(II) polypyridyl complexes have shown potential for light-triggered delivery of drugs with labile bonds, with possible activation by visible and infrared light, the latter being the ideal photodynamic therapy region [36,37]. Various Ru(II) polypyridyl complexes with luminescent capabilities have been investigated for applications as cellular probes and organelle stains [38]. The possible combination of the previously mentioned properties of Ru(II) propyridyl complexes for the design of a luminescent and photoreactive complex for double application in light-triggered drug delivery and cell imaging was recently investigated [39].

**Scheme 2:**
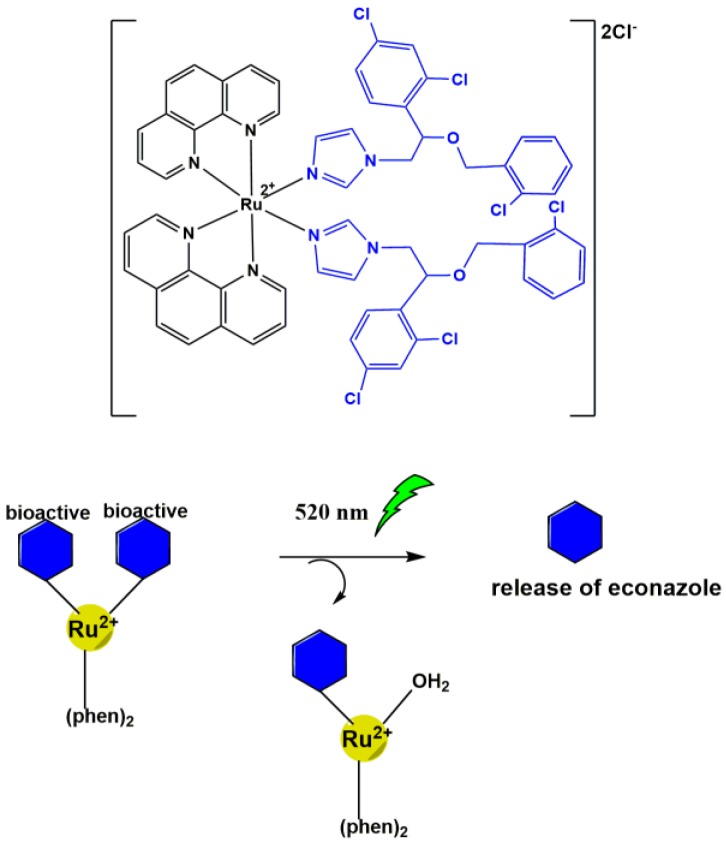
Activation of Ru(II) complex by green light with release of econazole.

Ru(II) polypyridyl complexes containing imidazole-based ligands have been demonstrated to reversibly modify the physicochemical properties of the coordinated drug. The antifungal econazole, which is currently being investigated as an anticancer agent, was used as an imidazole-based model drug due to its poor pharmacokinetics. A prodrug delivery system could therefore increase the effective dose of econazole, thereby positively impacting the effectiveness of this drug in treating various diseases [39]. Ru(II) complexes containing one or two econazole ligands were successfully prepared in a single step with moderate yield. The complex with two econazole ligands (Scheme 2) was an inactive and relatively safe prodrug of econazole, which could be activated by the use of green light. This complex displayed improved water stability and solubility, photo-selective toxicity, and favorable intracellular accumulation compared to the free drug. The low to moderate toxicity of this complex was observed in the absence of light but nanomolar IC_50_ values were noted upon combination of the complex with green light. The luminescence of this complex allowed for its visualization in live cells and drug release observation in actual time by the deactivation of the luminescence response. It must be noted, however, that after prolonged light activation the complex releases one bioactive molecule, which results in the non-fluorescent product, [Ru(o-phen)_2_(EZ)(H_2_O)]^2+^ (Scheme 2). Further research is therefore required to fully optimize these complexes for sustained delivery and imaging abilities. A Ru(II) complex with dual properties of photo-activated drug delivery and luminescence was therefore successfully obtained [39]. This approach could be extended for the delivery of drugs with similar chemical (imidazole-based) and pharmacokinetic properties to the model drug employed in this study, especially anticancer drug candidates that suffer from unfavorable pharmacokinetics and low tumor cell accumulation and selectivity.

From the literature, it is evident that ruthenium polypyridyl-linked neurochemical complexes provide rapid delivery and higher yields relative to other caged compounds. Moreover, a judicious choice of the ancillary ligand results in safer visible light stimulation as compared to UV light. However, in vivo design challenges such as adapting in vitro experiments and identifying the appropriate animal model to test the complexes must still be overcome to confirm the effectiveness of these compounds [39].

Chan and co-workers have recently investigated the use of photolabile Ru(II) complexes that can bind and release a purine model drug. It was demonstrated that the change of the ancillary ligand affected the stability and photolability of the complex [40]. The work undertaken emphasizes the versatility of the metal complex towards achieving optimized drug release behavior with minimal synthesis steps required. Nonetheless, in vivo studies should be undertaken.

## 4. Redox-Triggered Bioactive Release

**Scheme 3:**
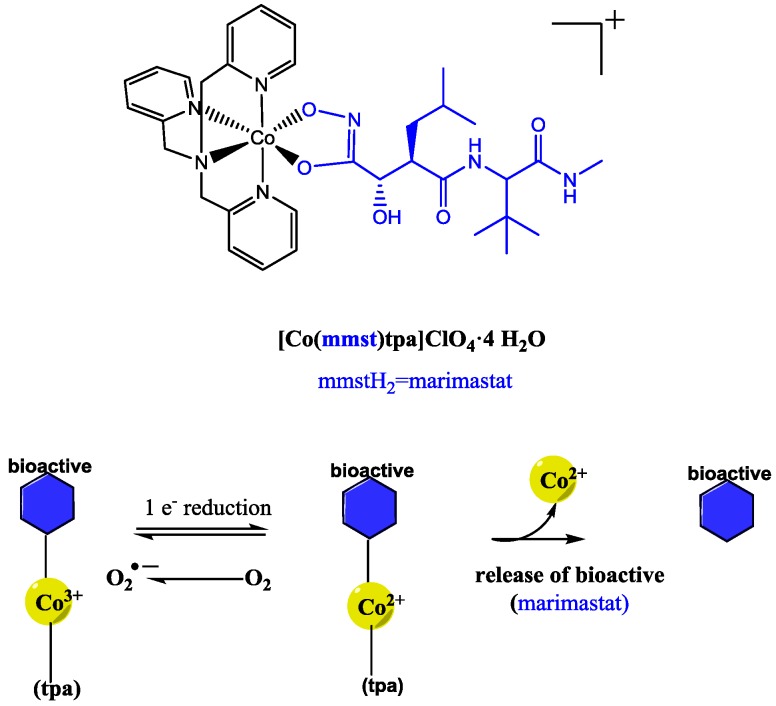
Proposed mechanism for the release of bioactive from a cobalt metal complex.

Compared to Co^3+^ complexes, which cannot undergo ligand exchange, complexes of Co^2+^ undergo simple ligand exchange, which endows these metal complexes with drug carrier functionality. Bioactive release usually ensues after ligand exchange by other nucleophiles in the biological milieu occurs. Moreover, both +2 and +3 cobalt species have been reported to possess no significant cell toxicity [41,42] at known doses. Pioneering work by Failes and co-workers therefore demonstrated that cobalt complexation to available “O” donor sites on metalloproteinase (MMP) inhibitors could result in two-fold design benefits [43]. Firstly, MMPs such as Marimastat^®^ have shown anti-cancer activity; however, this is limited in clinical application due to its poor oral bioavailability and proposed deactivation though interactions between the hydroxamic moieties in the presence of biomolecules [43]. Hence, complexation with Co(III)-tris(2-methylpyridyl)amine metal precursor (Co(III)-TPA) can prevent the Marimastat molecule from undergoing the abovementioned in vivo deactivation reactions. Secondly, the electrochemical properties of cobalt(III) could be used to selectively deliver Marimastat to hypoxic cells [44]. The delivery mechanism was proposed to occur in the anaerobic environment of tumor cells (highly negative redox potential), which promotes the reduction of cobalt(III) to the reactive cobalt(II), allowing the release of the bioactive via ligand exchange (Scheme 3) [45]. An in vitro MMP assay was used to establish the level of effectiveness of the cobalt(III) complex as a carrier for Marimastat and demonstrated the stability of that complex outside of a reducing environment, thereby preventing it from being deactivated prior to reaching the tumor site [45]. In vivo studies have verified the release of Marimastat; however, further work to investigate the in vivo fate of the cobalt metal carrier is necessitated.

Bustamante and co-workers aimed to develop new prototypes of a bioreductive drug by investigating the coordination of lawsone (2-hydroxy-1,4-naphtoquinone, HNQ) to transition metal ions [46]. They thus reported dimerization of lawsone upon reaction with Co(BF_4_)_2_·6H_2_O and *N*,*N*-bis(pyridin-2-ylmethyl)ethylenediamine (py_2_en), producing a bhnq^2−^ ligand (deprotonated form of HNQ) that ultimately coordinated to the Co(III) metal center in a single step to form the coordination complex [Co(bhnq)(py_2_en)]BF_4_^−^. The redox reactivity of the complex was observed in the presence of the reducing agent, ascorbic acid, under various oxygen concentrations and the results revealed that the reaction time between the metal complex and ascorbic acid is indirectly proportional to the concentration of oxygen [46]. This phenomenon could have been caused by either redox cycling, with Co(II) being oxidized back to Co(III) by oxygen prior to ligand dissociation, or competition between the Co(II) and the reducing agent for oxygen [46]. Furthermore, cyclic voltammetry experiments revealed that the metal complex obtained in this study can be easily reduced in the biological environment, and its Co(II) reduced form may be sufficiently inert to undergo redox cycling [46]. The dissociation of bhnq^2−^ after reduction with ascorbic acid is oxygen-dependent, suggesting the involvement of redox cycling [46]. This study showed the ability of HNQ to act as a model for the development of a bioreductive prodrug through metal complexation. Although there have been positive outcomes from the use of cobalt as a drug carrier, other factors in biological media that may affect the rate of ligand exchange to release the drug warrant further research.

## 5. Ion Activation-Triggered Release of Bioactive

**Figure 4:**
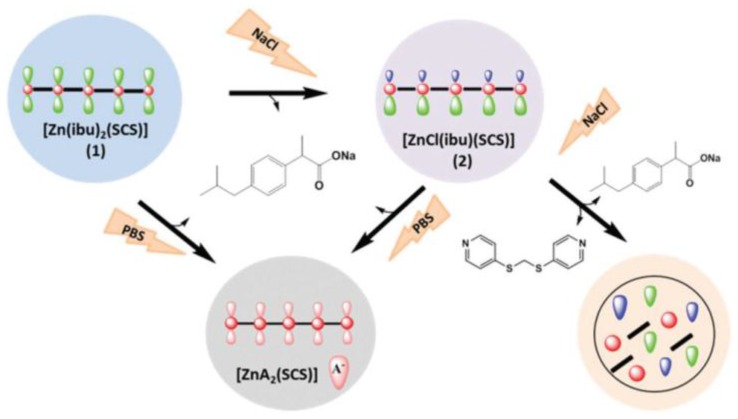
Graphical representation of the coordinating polymer based on (4-pyridylthio)methane (SCS) repeat units (black solid line), which is coordinated to the Zn-liganded Ibuprofen complex (Zn show in red and ibuprofen in green) followed by the anion exchange-triggered release of ibuprofen. Reproduced with permission from the Royal Chemical Society [47].

An example of the incorporation of both a polymeric support with stimuli-driven drug release was recently demonstrated by Lago and co-workers [47]. The fabricated polymer-loaded metal-liganded bioactive was prepared via a one-pot synthesis in water/ethanol solvent systems. Notably, the change in coordination geometry between the two complexes (1) and (2) depicted in Figure 4 resulted in an initial quick release followed by a slower dissociation release. The coordinated bioactive is released in a controlled manner by ligand exchange with phosphate anions due to competitive binding, i.e., the Zn metal center exhibits preferential binding to the phosphate ions.

## 6. Conclusions

In summary, metal-liganded bioactive stimuli-triggered release offers an innovative route for the development of controlled release vehicles. Based on the systems highlighted, several secondary routes have been pursued to create feasible and hierarchical designs that include bioMOFs, self-assemblies, and polymeric metal complexes. Preliminary proof of concept that metal–bioactive complex formation does occur and improves the performance of the bioactive is still required. Metal drug carriers have shown great potential for overcoming the limitations of marketed drugs by achieving improved pharmacokinetics with selective and controlled drug release of the parent drug.

However, there are further challenges faced in the translation of these systems into market-ready formulations. In fact, extensive studies should focus on the stability of the metal-liganded complex after in vivo administration and the in vivo fate of the metal carrier after drug release has occurred. Toxicity must be considered when working with metal complexes in medicine; however, efficient cell targeting in combination with stimuli-responsive release drug release systems would enable the safe delivery of the bioactive and will therefore negate bioaccumulation and toxicity issues. It must be noted that current drug delivery developments have placed an emphasis on macromolecular delivery vehicles (e.g., nanoparticles, dendrimers, liposomes), which present with challenges in achieving a well-defined particle size, and possess poor solubility, and retarded degradation with organ accumulation. Thus toxicity concerns such as blood clots, cardiovascular damage, and hypersensitivity reactions exist. Metal complexes do not notably affect the parent drug size and are small-molecule, well-defined drug delivery systems, and are thus exempt from a number of these limitations [8]. Furthermore, with the advancements in cellular imaging, a fluorescent functionalized metal-liganded carrier offers a strategy to investigate the pathways of metal trafficking. An understanding of these pathways will enable the development of optimized metal carriers that circumvent the perceived toxicity issues. Moreover, when metal-liganded bioactives are prepared they react in known stoichiometric ratios, and, coupled with full characterization, the exact metal content can be calculated. The resulting formulations are then systematically screened for cytotoxicity.

Nonetheless, the nascent technologies highlighted in the review could spur research into metal carriers as future novel drug delivery systems. A summary of these DDSs is presented in Table 1.

Based on the review of the varying stimuli responsive metal-liganded bioactive delivery systems, the following considerations should be implemented in future developments:

(a) In vitro studies must include metal-liganded bioactive release in a physiologically-simulated milieu to fully account for the stability, release mechanism, and possible free metal ion interactions.
(b) Possible inclusion of additional formulation science to overcome challenges faced in the physiological milieu (e.g., enteric targeting for oral systems).
(c) Evaluating whether the drug release kinetics observed translates to in vivo efficiency.
(d) Inclusion of in-depth in vitro cytotoxicity analyses in applicable cell lines and in vivo studies to mitigate the perceived risks involved in using metal carriers.

## Figures and Tables

**Table 1:** Summary of recent stimuli responsive metal-liganded bioactive drug delivery systems.

Metal	Bioactive	Highlights	Ref.
Zn(II)	Pipemidic acid	pH-triggered release of bioactive from the bioactive interpenetrated MOF.	[26]
Mg(II)	Olsalazine, Phenethylamine	Enhanced biocompatible carrier with programmed co-delivery of an interpenetrated bioactive and entrapped model drug.	[27]
Fe(III)	Doxorubicin	Biomimetic delivery of an anti-cancer drug.	[28]
Mg(II) & Ca(II)	Minocycline HCl	Sustained release triggered by pathology-induced tissue acidosis.	[29]
Ru(II)	Purine model drug 4-aminopyridine GABA	Versatile light-activated carrier for neuroactives and econazole ligands with addition of imaging potential. New reports for purine anti-cancer model drugs.	[30] [39] [40]
Co(III)	Metalloproteinase	Controlled bioreductive release demonstrated.	[43]
Zn(II)	Ibuprofen	Release is based on ligand exchange mechanisms.	[47]
